# Cyclosporine Treatment in Cats with Presumed Chronic Pancreatitis—A Retrospective Study

**DOI:** 10.3390/ani11102993

**Published:** 2021-10-19

**Authors:** Nina Hoeyrup, Thomas Spillmann, Linda Toresson

**Affiliations:** 1 Evidensia Specialist Animal Hospital Helsingborg, 254 66 Helsingborg, Sweden; nlhoeyrup@hotmail.com; 2 Department of Equine and Small Animal Medicine, Faculty of Veterinary Medicine, University of Helsinki, 00100 Helsinki, Finland; thomas.spillmann@helsinki.fi

**Keywords:** cyclosporine, cat, pancreatitis, Spec fPL

## Abstract

**Simple Summary:**

Chronic pancreatitis (CP) is a common disease in middle-aged to older cats. Cyclosporine, an immunosuppressive drug, has been suggested as an alternative treatment when other drugs that suppress inflammation are ineffective or unsuitable. However, no published studies have investigated its efficacy in cats with CP. The aim of this retrospective study was to evaluate the efficacy of cyclosporine as a treatment for pancreatitis in cats with presumed CP. All cats had a history and clinical signs suggestive of CP and blood samples showed abnormally high concentrations of feline pancreas-specific lipase (Spec fPL) on at least two occasions. This is common in CP. All cats were treated with cyclosporine for at least three weeks. Nineteen cats, aged 6.9–17.5 years, were included. Daily treatment with cyclosporine resulted in an improvement of serum Spec fPL concentrations which indicates that the pancreatic inflammation likely had improved. The study has several limitations, including different treatment durations and doses, lack of biopsies to confirm CP and the treatment effect was not compared with an untreated control group. Despite the limitations, our results suggest that cyclosporine treatment decreases serum Spec fPL concentrations and may be effective in the management of feline chronic pancreatitis.

**Abstract:**

Chronic pancreatitis (CP) is a common disease in middle-aged to older cats. Cyclosporine has been suggested as an alternative treatment when other immunosuppressive treatments are insufficient or contraindicated. However, no published studies have investigated its efficacy on feline CP. The aim of this retrospective study was to evaluate the efficacy of cyclosporine on supranormal serum feline pancreas-specific lipase (Spec fPL) concentrations in cats with presumed CP. Inclusion criteria were history and clinical signs suggestive of CP, serum Spec fPL concentrations above 5.3 μg/L (reference range 0–3.5 μg/L, equivocal range 3.6–5.3 μg/L) on at least two occasions and treatment with cyclosporine for at least three weeks. Serum Spec fPL was analyzed at Idexx Laboratories, Kornwestheim, Germany. Nineteen cats, aged 6.9–17.5 years (median 11.6), were included. No pancreatic biopsies were available. Median (range) serum Spec fPL concentration was 14.2 μg/L (6.1–43.3) at baseline and 6.7 μg/L (0.9–23.6) at follow-up. Cyclosporine treatment (5.0–7.9 mg/kg orally SID) was associated with a significant reduction in serum Spec fPL concentrations (*p* < 0.001) at follow-up after 23–206 days (median 35). Body weight decreased significantly between inclusion and follow-up (*p* = 0.013). Significant improvement of clinical signs could not be measured (*p* = 0.781). This study has several limitations, including unstandardized treatment length and dose, no control group and lack of pancreatic biopsies. Despite the limitations, our results suggest that cyclosporine treatment reduces supranormal serum Spec fPL concentrations in cats with presumed CP.

## 1. Introduction

Chronic pancreatitis (CP) is common in middle-aged to older cats [1]. Clinical signs of feline pancreatitis are often unspecific and subtle, including lethargy, anorexia, vomiting, weight loss and diarrhea [2,3,4,5,6]. Abdominal pain is common in dogs and humans with CP but is infrequently reported in cats [2,7,8]. The etiology of feline CP is unknown, but possible etiologies include immune-mediated disease, recurring acute pancreatitis (AP) leading to chronic changes or transmission of inflammation from surrounding abdominal organs such as the biliary system or the intestines [2,9,10,11,12]. However, the majority of cases are considered idiopathic [2]. CP is frequently identified on histopathology, even in apparently healthy asymptomatic cats [1].

Comorbidities are common and may include diabetes mellitus (DM), chronic enteropathies (CE), hepatitis, cholangitis and chronic kidney disease (CKD) [3,4,13,14,15,16]. Diabetes mellitus and exocrine pancreatic insufficiency (EPI) are believed to be possible sequelae to CP [10,12,17,18,19]. Cats with DM often have increased serum feline pancreas-specific lipase (Spec fPL) concentrations (Idexx Laboratories, Kornwestheim, Germany) or chronic pancreatitis on histopathology and it is possible that CP may contribute to reduced glycemic control [19,20,21,22].

Histopathology is the gold standard for diagnosing feline CP with fibrosis, cysts and lymphocytic inflammation as the dominating features [1,2,9,13]. However, many cats with suspected CP are not optimal candidates to undergo general anesthesia and biopsying. Furthermore, as the pancreatic changes are often focal, biopsies may miss the disease processes [1,2,13]. Serum Spec fPL is a species-specific, enzyme-linked immunosorbent assay that measures lipase produced by pancreatic tissue [23]. Measurement of serum Spec fPL concentrations is a non-invasive alternative in diagnosing feline pancreatitis, with a reported specificity of 63–100% and sensitivity of 54–100% [13,24]. However, the reported sensitivity is lower in mild or chronic pancreatitis compared to acute or moderate to severe pancreatitis.

There is currently no gold standard treatment of feline CP, though corticosteroids are frequently used [2]. If corticosteroid treatment is insufficient or contraindicated, such as in cats with DM, cyclosporine is a possible alternative [2]. Cyclosporine is an immunosuppressive drug that acts by inhibiting calcineurin and thereby reduces T cell activity and cell-mediated immunity [25].

In cats, cyclosporine is licensed for chronic allergic dermatitis and off-label uses include CE, immune-mediated anemia, eosinophilic granuloma and stomatitis, pemphigus foliaceus and prevention of tissue rejection after renal transplantation [25,26,27,28,29,30]. There are no published studies on the use of cyclosporine as treatment for CP or CE in cats. Adverse effects in cats are common and may include vomiting, hypersalivation, diarrhea, anorexia, weight loss, lethargy and gingival hyperplasia [31,32,33,34,35,36]. Cyclosporine may increase the risk of infection with or reactivation of latent Toxoplasma gondii infection and contribute to the development of lymphoma [31,33,35,37,38,39].

In dogs, cyclosporine has been used in the treatment of CE and protein-losing enteropathy [40,41,42]. Steiner and Huber [21] published a case report on a dog with atopy, DM and a supranormal serum canine pancreas-specific lipase (Spec cPL) concentration. The dog was treated with cyclosporine to control the atopy. During treatment, a better glycemic control was achieved and the serum Spec cPL concentration was normalized. In mice, cyclosporine has been shown to have an anti-inflammatory effect on experimentally-induced autoimmune pancreatitis [43]. The suggested effect of cyclosporine was by inhibiting T effector cell activation and proliferation, thus reducing the T cell dominated pancreatic inflammation.

The purpose of this retrospective study was to compare serum Spec fPL concentrations in cats with presumed CP before and during treatment with cyclosporine, thereby assessing the potential use of cyclosporine in the treatment of feline CP.

## 2. Materials and Methods

The medical records of Evidensia Specialist Animal Hospital, Helsingborg, Sweden (ESAHHS), were searched for cats treated with cyclosporine for suspected CP during 2013–2019. Inclusion criteria were suspected CP, defined as serum Spec fPL concentrations >5.3 μg/L (reference interval 0–3.5 μg/L, equivocal range 3.6–5.3 μg/L) on two or more occasions at least three weeks apart, combined with a clinical suspicion of CP based on clinical signs compatible with pancreatic disease and/or diabetes mellitus. Clinical signs regarded as possibly related to chronic pancreatitis were vomiting, lethargy, hyporexia, weight loss and abdominal pain. Additional inclusion criteria were sufficiently detailed clinical records, treatment with cyclosporine for a minimum of three weeks and a follow-up visit with a serum Spec fPL measurement during treatment. Cats receiving corticosteroids at inclusion were required to have an increased Spec fPL concentration, despite corticosteroid treatment, prior to inclusion. Exclusion criteria were lack of owner compliance, tapering/withdrawal of cyclosporine before the follow-up, starting additional immunosuppressive treatments or increasing the dose of concurrent maintenance corticosteroid therapy before the follow-up.

Information gathered from the clinical records included age, breed, gender, body weight (BW), body condition score (BCS) and comorbidities. Clinical signs, diet and medical treatments related to gastrointestinal or pancreatic disease were recorded at inclusion and until follow-up. Regarding cyclosporine, brand and formulation, treatment length and dose were recorded. Information was also gathered on clinical pathology, including serum Spec fPL concentrations, serum feline Trypsin-Like Immunoreactivity (fTLI) concentrations, serum cobalamin and folate measurements, ultrasonographic imaging of the abdomen and histopathological analysis of gastrointestinal and liver biopsies. Clinical signs were retrospectively quantified using the Feline Chronic Enteropathy Activity Index (FCEAI) developed by Jergens et al., 2010 [44]. A FCEAI score was calculated for the last visit prior to inclusion and for the follow-up visit. Adverse effects and outcome were recorded for the entire cyclosporine treatment period and not only for the study period from inclusion to follow-up.

Serum Spec fPL concentrations were analyzed by Idexx Laboratories, Kornwestheim, Germany. Additional clinical chemistry analyses were performed at ESAHHS, referring clinics or referral laboratories. The histopathological examinations of gastrointestinal biopsies were performed by pathologists at BioVet AB, Sollentuna, Sweden, the National Veterinary Institute, Uppsala, Sweden, or Idexx Laboratories, Kornwestheim, Germany. Ultrasonographic examinations were performed by veterinarians with a minimum of three years of experience with ultrasonography and, on one occasion, a Swedish specialist in diagnostic imaging.

### Statistical Analysis

Statistical analysis was performed using GraphPad Prism 8.0 (GraphPad Software). The effect of cyclosporine treatment on Serum Spec fPL concentrations was tested for normality using the D’Agostino and Pearson test and was analyzed for statistical significance using the Wilcoxon matched pairs signed rank test. Body weight, BCS and FCEAI at baseline and follow-up were also tested for normality using the D’Agostino and Pearson test. Body weight and BCS were analyzed for statistical significance with the paired *t*-test and FCEAI with the Wilcoxon matched-pairs signed rank test. Statistical significance was determined as a *p*-value < 0.05.

## 3. Results

### 3.1. Baseline Data and Clinical Signs

A total of 19 cats were prescribed cyclosporine for suspected CP during 2013–2019 and matched the inclusion criteria. At inclusion, the median (range) age was 11.6 years (6.9–17.5). Domestic short hair was the most common breed (Table 1). Sixteen cats were neutered males and three neutered females. Information on BW was available for all cats at inclusion and for 16/19 cats at follow-up, and BCS was available for 16/19 cats on both occasions. Median BW at inclusion was 5.7 kg (3.0–7.2) and median BCS was 6/9 (3/9–8/9). At the follow-up, median BW was 5.6 kg (2.7–7.0) and median BCS was 5/9 (3/9–7/9). Body weight had decreased in 12 cats. The differences between inclusion and follow-up were statistically significant for BW (*p* = 0.013) and non-significant for BCS (*p* = 0.118). In 9/12 cats with loss of BW during the study period, weight loss was also reported at inclusion.

Clinical signs at inclusion were weight loss (n = 12), lethargy (n = 9), hyporexia (n = 8), vomiting (n = 8), diarrhea (n = 5) and constipation (n = 3). In six cats, abdominal pain was identified by the treating clinician during physical examination. In 13/19 cats, the primary clinical concern was clinical signs compatible with CP and/or CE, e.g., vomiting, hyporexia, weight loss, lethargy and/or abdominal pain. In the remaining six cats, the primary concern was DM, however two of these also had occasional loose stool or poor appetite.

FCEAI scores could be calculated for 12/19 cats due to incomplete clinical records. Of these 12 cats, 4 had DM as their main complaint. The median (range) baseline FCEAI score was 2.0 (0–6.0). At follow-up, the median FCEAI score was 1.5 (0–7.0). There was no significant difference in FCEAI between baseline and follow-up (*p* = 0.781).

### 3.2. Comorbidities

Comorbidities at study inclusion were common and included suspected or confirmed CE (n = 14), CKD (n = 9), DM (n = 6) and suspected or confirmed hepatobiliary disease (n = 4) (Table 1 and Table 2). Chronic enteropathy was confirmed by histopathology in six cats (Table 2) although ileal biopsies were not available to rule out alimentary lymphoma. In an additional eight cats, chronic enteropathy was suspected based on subnormal or low-normal serum cobalamin concentrations (n = 8) and ultrasonographic signs of CE (n = 2). An ultrasonographic suspicion of CE was based on thickening of the small intestinal muscularis layer and an overall assessment by the ultrasonographic examiner [45,46]. Eight months after starting cyclosporine treatment, and one month after the follow-up visit, one of the cats with confirmed CE was diagnosed with lymphoma in the mesenteric lymph nodes. The diagnosis was based on histopathology of surgical biopsies from those lymph nodes.

Staging of CKD, according to the International Renal Interest Society (IRIS) guidelines, was possible for 15/19 cats at inclusion and for 13/19 cats at follow-up [47]. Nine cats had CKD at inclusion with an IRIS stage of 1 (n = 4) or 2 (n = 5) and at follow-up, eight cats had IRIS stage 1 (n = 1) or 2 (n = 7). Two cats had progressed from IRIS stage 1 to stage 2 and one cat with IRIS stage 1 at inclusion could not be staged at follow-up. All six cats with DM were diagnosed prior to study inclusion, though a seventh cat developed DM seven months after starting cyclosporine treatment. This cat was euthanized following the diagnosis of DM. Five of the six cats with DM were insulin-dependent at inclusion and two went into remission during cyclosporine treatment. The sixth cat with DM had gone into remission a month before study inclusion but DM recurred seven months after starting cyclosporine treatment.

### 3.3. Medical History and Diet

All cats received commercial diets during the study period (Table 3). Most cats received several different diets, including one cat fed a commercial raw food diet. Between inclusion and follow-up, novel types of canned food or diabetic dry food were added to the diet of 4/19 cats, but otherwise diets remained unchanged.

Before inclusion, 14/19 cats received corticosteroids for pancreatic and/or gastrointestinal disease. None of the remaining five cats were treated with corticosteroids between inclusion and follow-up. Thirteen cats were still treated with corticosteroids at inclusion. The cats had received corticosteroids for 12–3286 days (median 224) before starting cyclosporine treatment. Between inclusion and follow-up, the corticosteroid dose was tapered in 3/13 cats and suspended in 1/13. The corticosteroid dose was not increased in any of the cats during this time period. Miscellaneous concurrent treatments for pancreatic and/or gastrointestinal disease are listed in Table 3.

### 3.4. Clinical Chemistry

Serum cobalamin concentrations were analyzed at inclusion in 12 cats and were within the reference range in all cats. However, three cats had serum cobalamin concentrations within 100 units from the lower reference limit (Table 4). At follow-up, serum cobalamin concentrations were measured in 3 cats and subnormal in 1/3 (Table 4). None of the cats had supranormal serum cobalamin concentrations at inclusion or follow-up. In addition to the four cats with subnormal or low-normal serum cobalamin concentrations during the study period, eight cats had subnormal or low-normal concentrations before (n = 3) or after (n = 5) the study period. In total, 12/19 cats had subnormal or low-normal serum cobalamin concentrations, of which four had histopathologically confirmed CE. Serum folate concentrations were analyzed in 11 cats at inclusion (n = 9) or follow-up (n = 2), and all measurements were within the reference interval (RI). Serum fTLI concentrations were analyzed in one cat during the study period and in four other cats before (n = 2) or after (n = 2) the study period. All fTLI measurements were within the RI. Serum creatinine concentrations were analyzed at inclusion in 13 cats and at follow-up in 12 cats. Serum creatinine concentrations were supranormal in 2/13 at inclusion and in 3/12 at follow-up, including one of the cats with a supranormal concentration at inclusion.

### 3.5. Ultrasonography

Ultrasonography of the pancreas was performed in all 19 cats. In total, 14/19 cats were diagnosed with an abnormal pancreas on ultrasonographic examination on at least one occasion. Reported ultrasonographic abnormalities indicating possible pancreatitis included a hypoechoic, hyperechoic, heterogenic, prominent or enlarged pancreas, increased peripancreatic echogenicity and a dilated pancreatic duct [2,48].

In 10 cats, ultrasonography of the pancreas was performed during the study period and the pancreas appeared abnormal in 4/10 (Table 5). In 3/6 cats with an ultrasonographically normal pancreas during the study period, the pancreas appeared abnormal on ultrasonographic examinations prior to or after the study period. In the remaining nine cats, ultrasonography of the pancreas was performed prior to inclusion (n = 4) or after follow-up (n = 5) and the pancreas appeared abnormal in seven of these cats.

Results from ultrasonographic examination of the gastrointestinal tract and hepatobiliary system during the study period is available in Table 5. CKD was suspected on ultrasonography in four cats at study inclusion (n = 3) or follow-up (n = 1).

### 3.6. Cyclosporine Treatment

The median (range) cyclosporine dose at inclusion was 7.0 mg/kg (5.0–7.6) administered once daily. After follow-up, cyclosporine treatment was adjusted in all cats. The dose was decreased in 10/19 cats, increased in 2/19 and cyclosporine was withdrawn in 7/19. Of the cats that underwent dose tapering, the median time from follow-up until tapering was 11 days (0–152). After tapering, 8/10 cats were treated with cyclosporine every other day, 1/10 every third day and 1/10 received a reduced daily dose. The dose was increased in 2/19 cats as they had received a cyclosporine dose below the standard recommendation of 7.0 mg/kg. Three of nineteen cats were excluded from further statistical analysis after tapering (n = 2) or withdrawal (n = 1) of cyclosporine as they had no further follow-up. In the remaining eight cats with a tapered dose, the median dose after tapering was 3.1 mg/kg/day (2.2–4.7). In 7/8 cats, the dose was increased again after tapering or withdrawal due to increasing supranormal serum Spec fPL concentrations at control visits. Four of the seven cats had also experienced worsening clinical signs, including vomiting (n = 3), lethargy (n = 2), constipation (n = 1) and diarrhea (n = 1). Two of seven cats were excluded from further statistics as they had no follow-up after dose increase. The median dose of the remaining five cats was 6.9 mg/kg/day (6.0–8.3).

At inclusion, 14 cats received an oral cyclosporine solution and 5 received capsules. Administered brands at inclusion were Atopica (Elanco Denmark, Ballerup, Denmark) (n = 17) and Sporimune (Dechra Veterinary Products AB, Upplands Vaesby, Sweden) (n = 2).

Information on whether the owners administered cyclosporine with or without food was unavailable.

### 3.7. Serum Spec fPL Concentrations

**Figure 1:**
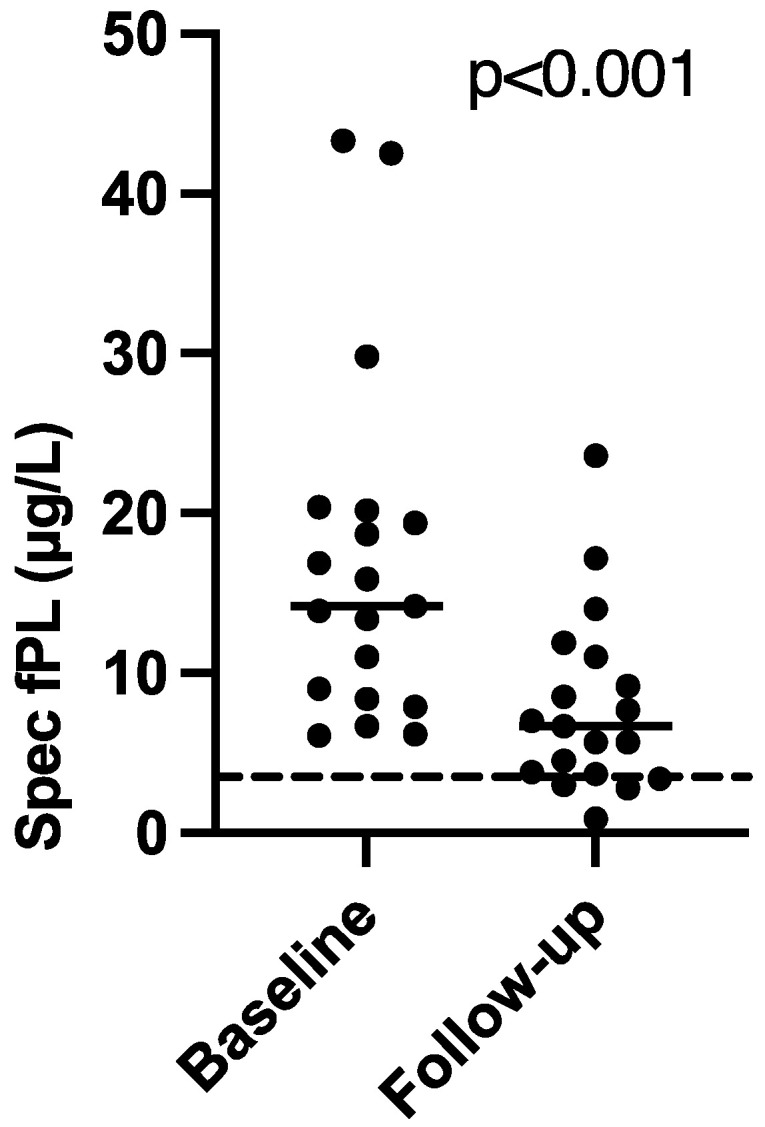
Comparison of serum Spec fPL concentrations at baseline and follow-up in 19 cats with presumed chronic pancreatitis treated with cyclosporine. Short horizontal lines represent median, long horizontal stippled line represents the upper limit of the serum Spec fPL reference interval (3.5 µg/L).

The majority of the cats were diagnosed with presumed CP several months before study inclusion. The median (range) time from the first supranormal serum Spec fPL concentration to inclusion was 103 days (7–1605). The median (range) serum Spec fPL concentration at inclusion was 14.2 μg/L (6.1–43.3). At follow-up, the median serum Spec fPL concentration had decreased significantly to 6.7 μg/L (0.9–23.6) (*p* < 0.001) (Figure 1). Sixteen cats had a decrease in serum Spec fPL concentration (serological responders) and three cats had an increase (serological non-responders). The median time from inclusion to follow-up was 35 days (23–206).

**Figure 2:**
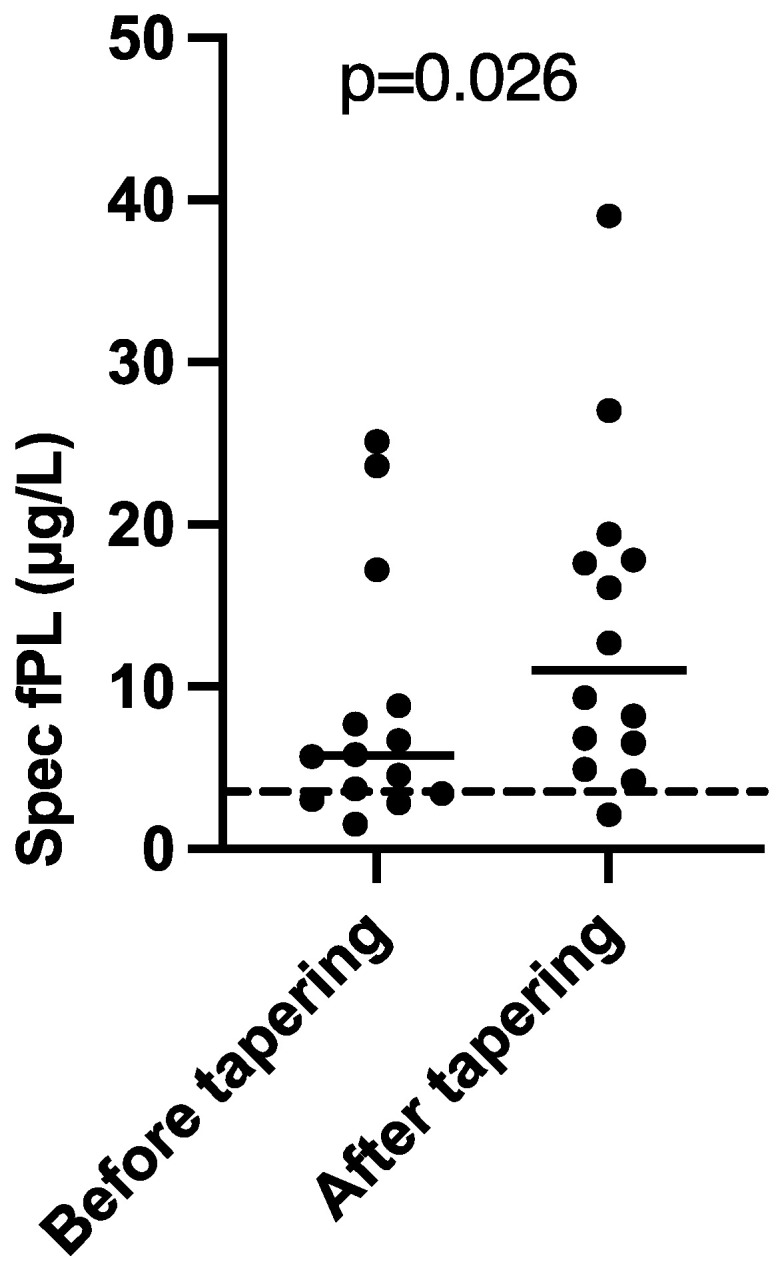
Comparison of serum Spec fPL concentrations before tapering/withdrawal and at follow-up in 14/19 cats with presumed chronic pancreatitis treated with cyclosporine. Short horizontal lines represent median, long horizontal stippled line represents upper limit of the reference interval (3.5 µg/L).

In 14 cats with sufficient record data for statistical calculations (11 serological responders, 3 serological non-responders), cyclosporine treatment was tapered (n = 8) or withdrawn (n = 6) after follow-up. This was associated with a statistically significant increase in serum Spec fPL concentrations (*p* = 0.026). Before tapering/withdrawal, the 14 cats had a median (range) serum Spec fPL concentration of 5.8 μg/L (1.5–25.1) which increased to 11.0 μg/L (2.1–39.0) after dose reduction (Figure 2). Serum Spec fPL concentrations had increased in 10/14 cats, including 1/3 initial serological non-responders, and decreased in 4/14 cats, including 2/3 serological non-responders. The median time from tapering/withdrawal to follow-up was 61 days (13–120).

**Figure 3:**
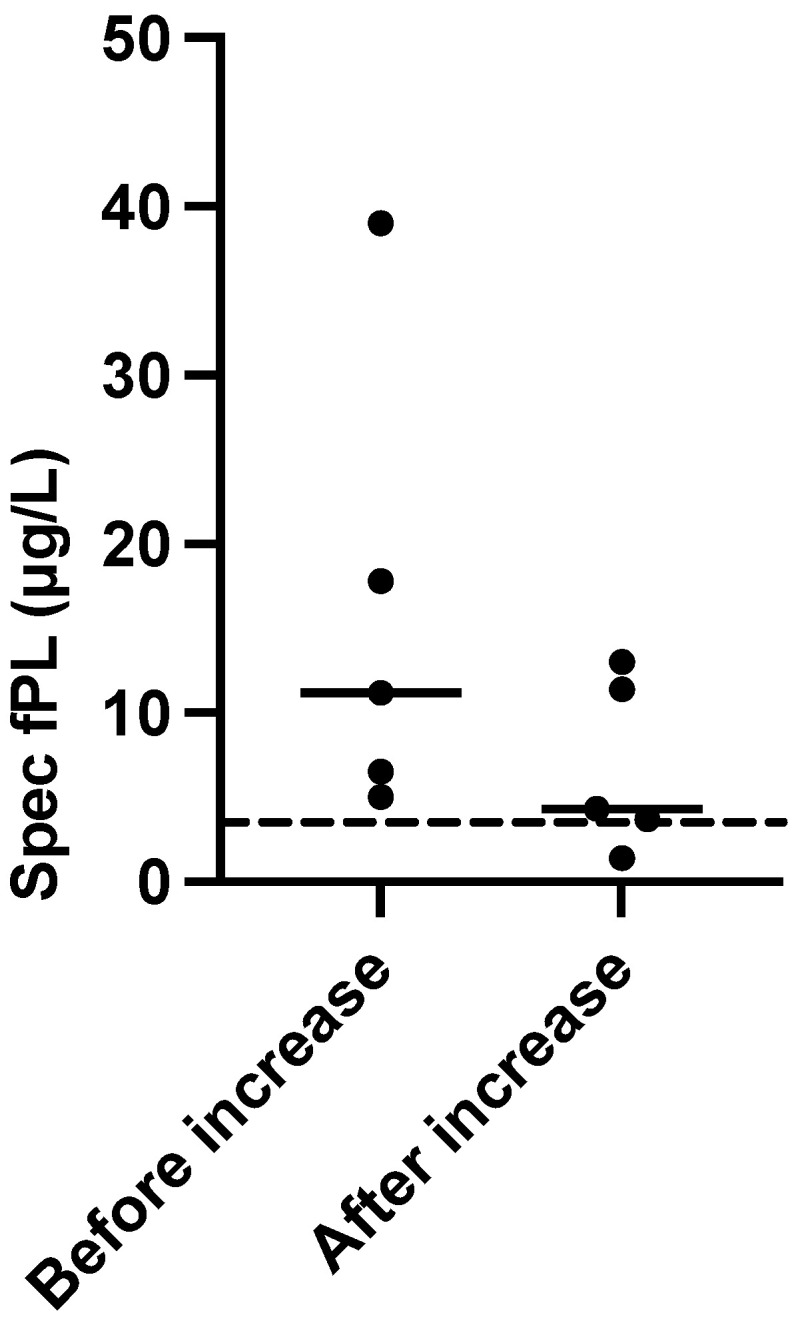
Comparison of serum Spec fPL concentrations before dose increase and at follow-up in 5/19 cats with presumed chronic pancreatitis treated with cyclosporine. Short horizontal lines represent median, long horizontal stippled line represents the upper limit of the serum Spec fPL reference interval (3.5 µg/L).

In five cats, the cyclosporine dose was again increased after tapering/withdrawal. Before the increase, the median (range) serum Spec fPL concentration was 14.5 μg/L (5.0–39.0) which decreased to 7.6 μg/L (1.4–13.0) after dose adjustment. The median time from dose increase to follow-up was 59 days (30–79). The serum Spec fPL concentrations had decreased in all five cats, including one initial serological non-responder. The number of cats was too small for statistical analysis (Figure 3).

### 3.8. Adverse Effects

Adverse effects during the entire cyclosporine treatment period were reported in 11/19 cats and included vomiting (n = 4), hypersalivation (n = 3) or tremor (n = 1) after cyclosporine administration, gingival hyperplasia (n = 1), matted fur (n = 1), lethargy (n = 1) and diarrhea (n = 1). Of the four cats with reported vomiting, one cat vomited several times on treatment day three but tolerated the medication well after cyclosporine was withdrawn and then reintroduced gradually. Another cat vomited once directly after the cyclosporine capsule was administered and subsequently became difficult to medicate. A third cat tolerated cyclosporine very well during several months but started to vomit much more frequently after five months. The owner started administering maropitant prior to cyclosporine during periods of vomiting which seemed to help. Treatment with cyclosporine was continued. The fourth cat had increased frequency of vomiting during cyclosporine treatment compared to prior to treatment. One cat was diagnosed with lymphoma during cyclosporine treatment. Measurements of serum creatinine concentrations during cyclosporine treatment were not performed at standardized time intervals. Seven cats developed supranormal serum creatinine values with a median increase of 48% (range 20–108%) from inclusion to the first supranormal concentration measured 24–1957 days (median 133 days) after cyclosporine treatment started. In one cat, pre-existing supranormal serum creatinine had increased by 12% when measured 32 days after inclusion. In one cat with diarrhea after starting cyclosporine, the diarrhea resolved when the medication was withdrawn and then reintroduced gradually. Six of nineteen cats disliked the taste of cyclosporine and/or hid at the time of administration or immediately after. Three cats were switched from oral solution to capsules to reduce adverse effects in connection with cyclosporine administration, and for 2/3 a reduction in adverse effects was reported.

### 3.9. Outcome

Treatment with cyclosporine was withdrawn in 11/19 cats due to adverse effects (n = 7), lack of clinical effect (n = 3) or cost (n = 1). Two cats with DM went into remission five and five and a half months, respectively, after cyclosporine treatment commenced. When remission was suspected, the insulin doses were gradually reduced over two to five months and none of the cats relapsed. Cyclosporine treatment was continued in both cats after remission. At the time of writing, three cats were still alive, two cats were lost to follow-up and 14 had been euthanized. Known reasons for euthanasia included clinical signs related to gastrointestinal and/or pancreatic disease (n = 5), including the cat with lymphoma in the mesenteric lymph nodes, DM (n = 2), kidney failure (n = 1), respiratory disease (n = 1), respiratory disease and otitis media (n = 1) and liver failure (n = 1). For 12 cats, the date of euthanasia was available. In those cats, the median (range) time from starting cyclosporine treatment to euthanasia was 380 days (60–1199), and the median age at euthanasia was 14.2 years (range 10.2–17.5). Of these 12 cats, 6 were still treated with cyclosporine at time of euthanasia. The three cats still alive at the time of writing were still treated with cyclosporine and had received cyclosporine for a median of 1330 days (1207–2587).

## 4. Discussion

In this retrospective study, CP was presumed in 19 cats based on clinical signs, elevated serum Spec fPL concentrations and ultrasonographic findings. Serum Spec fPL concentrations were compared before and after cyclosporine treatment was initiated. Cyclosporine treatment resulted in a significant reduction in supranormal serum Spec fPL concentrations. A subsequent dose tapering or withdrawal of cyclosporine in 14/19 cats resulted in a significant increase in serum Spec fPL concentrations. The cyclosporine dose was again increased in 5/14 cats, which was associated with a subsequent reduction in serum Spec fPL concentrations. When using the search engines PubMed, Ref Seek and Google Scholar, no reports on treatment of feline pancreatitis with cyclosporine have been found. Consequently, this is the first report on treatment of presumed feline CP with cyclosporine.

Three cats were alive at the time of writing, and all were still treated with cyclosporine. It could be speculated that cyclosporine treatment, if tolerated, may increase longevity in cats with CP. However, three cats is too small a number to draw any conclusions.

The exact mechanism by which cyclosporine may reduce pancreatic inflammation is unknown, but suggested mechanisms are reduction in T lymphocyte activity and interleukin release and thereby suppression of the cell-mediated immune response [43]. A study by Schwaiger et al. [43] examined the effect of cyclosporine, rapamycin and azathioprine on experimentally-induced autoimmune pancreatitis in mice. Mice treated with cyclosporine had significantly less inflammatory infiltration and significantly reduced destruction of pancreatic acinar cells compared to placebo and azathioprine. In the study, the pancreatic inflammatory infiltrate was dominated by T lymphocytes. Lymphocytes are also reported to be the primary inflammatory cell type in feline CP [1]. Thus, reduction in T lymphocyte activity could explain how cyclosporine may improve pancreatic inflammation.

A very large number (16/19 or 84%) of the cats in this study were neutered males and only three (16%) were neutered females. Feline CP is not usually regarded as having any gender disposition [2]. Similar to the present study, Schnauß et al. [3] reported that 17/21 (81%) of cats with a very high likelihood of having pancreatitis were male but other studies have not shown a similarly skewed gender representation.

Currently, it is unknown whether the choice of diet has a major impact on CP in cats [2]. Dietary recommendations in this study were commonly based on concurrent diseases such as DM and CE. Structured dietary management was not common, and the owners generally had difficulties maintaining their cats on strict diets. Many of the cats had already undergone dietary trials and adjustments before starting cyclosporine treatment. There were no major changes in diet during the study period except for the addition of canned food or diabetic dry food to the existing diet in four cats. Thus, it is difficult to assess a possible influence of diet on outcome, clinical signs and the development of serum Spec fPL concentrations.

The study did not show a clear improvement of clinical signs during cyclosporine treatment. The poor correlation between reduction in supranormal serum Spec fPL concentrations, improvement of clinical signs and FCEAI scores highlights the difficulty in assessing the clinical relevance of supranormal serum Spec fPL concentrations. The retrospective nature of the study markedly reduced the availability and accuracy of information on clinical signs, making quantification and statistical comparisons of clinical signs more difficult. Only 12 cats had sufficient data available for calculating FCEAI scores, including 4 cats with DM as their primary concern. As the cats with DM generally had fewer clinical signs, this may also have contributed to the non-significant development of clinical signs. However, the discrepancy may also be due to the often rather subtle clinical signs of feline CP which may be missed by the owners. Norsworthy et al. (2013) [49] reported that cat owners frequently interpreted clinical signs of CE, such as vomiting, as normal. Thus, existing clinical signs may have been underreported in the present study. Further possible causes of the lack of improvement in clinical signs are possible relations to comorbidities or that the supranormal serum Spec fPL concentrations did not reflect an actual pancreatic inflammation. In one report, 16/23 cats with CE had increased serum feline pancreatic lipase immunoreactivity (PLI) concentrations. Whether this increase was associated with CP remained uncertain due to the lack of pancreatic histopathology [50]. As previously mentioned, the diagnosis of CP was not confirmed by histopathology in our study either, increasing the risk that there were other causes for the clinical signs. However, 14/19 cats with repeatedly increased serum Spec fPL concentrations also had an abnormal pancreas on ultrasonographic examination on at least one occasion. This increases the probability that CP was associated with the reported clinical signs. The remaining five cats with an ultrasonographically normal pancreas had at least three supranormal serum Spec fPL concentrations over a period of at least five months. Prospective studies are warranted to properly assess the clinical and histopathological effects of cyclosporine treatment in cats with confirmed CP.

Even though positive clinical effects of cyclosporine treatment were difficult to assess in this study, a clinical benefit was obvious in one cat. The cat had DM, suspected CP and CE, based on clinical signs, ultrasonographic findings and an unmeasurable subnormal serum cobalamin concentration. During treatment with cyclosporine, the cat’s serum Spec fPL concentrations normalized. The owner subsequently misunderstood the instructions from the treating clinician (L.T.) and stopped cyclosporine treatment. At that time, the cat was bright and alert with a good appetite. Two months after cyclosporine treatment was suspended, the cat had gradually become hyporectic and lethargic, lost 12% of its BW and serum Spec fPL concentration had increased from 3.4 to 6.4 µg/L. The cat had developed very poor glycemic control on the same dose of insulin as before withdrawal of cyclosporine. When cyclosporine was re-introduced, clinical status was reversed and the DM stabilized, although the insulin dose had to be increased twice.

Chronic pancreatitis has been suggested as a possible reason for unstable DM in cats [19,22]. Monitoring of glycemic control varied significantly between the cats, making assessment of cyclosporine’s effect on glycemic control difficult. Two of five cats with insulin-dependent DM at inclusion subsequently went into remission and cyclosporine may have contributed to this. In a third cat, described in the previous section, cyclosporine appeared to improve glycemic control. This cat was started on cyclosporine during diabetes remission, but it did not prevent the recurrence of DM. However, when the owner later stopped cyclosporine treatment, glycemic control went from excellent to poor on the same dose of insulin. It could thus be speculated that cyclosporine treatment had a positive effect on glycemic control in 3/6 cats with DM at inclusion. In a case report of a dog with DM, cyclosporine not only improved supranormal serum Spec cPL concentrations but was also associated with significantly improved glycemic control in a dog [21]. However, it is unknown whether the potential effect was by reducing pancreatic inflammation or if cyclosporine affected the DM more directly. Studies in people have shown that cyclosporine may cause diabetic remission in patients with type 1 DM by preserving β-cell function though remission was usually transient [51,52]. Reduced β-cell function and insulin secretion is usually involved in the disease process of feline DM as well [53]. Thus, the potential positive effect of cyclosporine on glycemic control in the cats with DM and presumed CP in our study may be either indirect by reducing pancreatic inflammation and insulin resistance or direct by preserving β-cell function and insulin secretion.

In humans treated with cyclosporine after organ transplantation, cyclosporine has been shown to cause hyperglycemia and DM, despite the above mentioned study showing a potential positive effect in type 1 DM [54]. Similarly, cyclosporine has been shown to cause hyperglycemia in rats [55]. Both in people and in rats, decreased insulin secretion as well as increased insulin resistance are believed to contribute to the hyperglycemia [54,55]. Further studies are needed to determine if cyclosporine may have a positive effect on DM in cats with CP or whether the suggested positive influence of cyclosporine on glycemic control in this study is merely incidental.

Adverse effects and difficulties in administration of cyclosporine were common, potentially causing poor owner compliance. However, vomiting was interpreted as an adverse effect in four cats, but this could also have been caused by a flare-up of CP as well.

The cost of cyclosporine also deterred one owner from continuing treatment. In several cats, adverse effects in connection with administration of cyclosporine were alleviated by switching from liquid formula to capsules or by administering an antiemetic shortly before cyclosporine administration. One cat with confirmed CE was diagnosed with lymphoma in the mesenteric lymph nodes eight months after starting cyclosporine treatment. Cyclosporine may have contributed to this as it has previously been suspected of contributing to the development of lymphoma in cats [31,39]. During the study period, two cats progressed from IRIS stage 1 at inclusion to stage 2 at follow-up. Though CKD is very common in older cats, cyclosporine treatment may have contributed to the development or progression of CKD. Cyclosporine is considered nephrotoxic in humans though initially the effect is usually reversible [52].

There was a statistically significant weight loss among the cats between inclusion and follow-up. Weight loss was reported in 12/19 cats at inclusion, and 9 had continued weight loss between inclusion and follow-up. In an additional three cats, BW had decreased between inclusion and follow-up. Heinrich et al., 2011 [31] reported weight loss in 8/50 cats treated with cyclosporine for allergic dermatitis, though it was unknown if the cause was comorbidities and/or the cyclosporine treatment. The larger proportion of cats with weight loss during the study period in our study is likely also due to preexisting comorbidities, though it is unknown if nausea associated with cyclosporine administration exacerbated this significantly.

Chronic enteropathy was confirmed by histopathology in 6 cats but was suspected by the treating clinician in an additional 8/13 remaining cats. All eight cats had subnormal or low-normal serum cobalamin concentrations and two cats also had ultrasonographic findings suggestive of CE. Hypocobalaminemia could have been caused by concurrent CE, undiagnosed EPI or intestinal lymphoma [56]. Bailey et al., 2010 [50] compared cats with confirmed CE with or without supranormal serum fPL concentrations and found that cats with supranormal serum fPL concentrations were significantly more likely to have hypocobalaminemia. Serum fTLI concentrations were measured once in 5/19 cats. Two cats had fTLI analyzed by the referring vet prior to analysis of serum fPL concentrations. In three cats, fTLI were measured at a later time point since the cats continued to lose weight and EPI can be a consequence of chronic pancreatitis and subsequent pancreatic atrophy [10,18]. EPI may have been missed in some of the other cats and could contribute to weight loss, a worse outcome and lack of improvement of clinical signs which are often similar to those in CP [18]. However, since serum Spec fPL concentrations were supranormal, some of the exocrine pancreatic tissue must still be preserved and functioning. Thus, cobalamin deficiency was more likely an effect of CE than EPI.

The majority of the cats had been treated with corticosteroids before starting cyclosporine treatment. Though 13 cats were still treated with corticosteroids at inclusion, it is unlikely that this treatment was a major contribution to the improvement of serum Spec fPL concentrations as no satisfactory improvement in Spec fPL concentrations had occurred previously during treatment. Only one cat had been treated with corticosteroids for less than six weeks prior to inclusion. The cat developed DM after 12 days of corticosteroid treatment and the treatment was therefore suspended. One cat began treatment with prednisolone after the follow-up visit. Due to adverse effects, cyclosporine was paused, and prednisolone introduced. During the following three weeks, the cat’s appetite decreased markedly from normal to only accepting a frozen raw food diet despite being treated with a daily prednisolone dose of 1.8 mg/kg. Spec fPL increased from 1.5 to 17.8 μg/L during this time (results included in Figure 2). Cyclosporine was gradually re-introduced, and prednisolone tapered. Three and a half months later, cyclosporine was administered at a daily dose of 6.1 mg/kg, prednisolone had been withdrawn and the cat was accepting its original diet again. Serum Spec fPL concentrations had decreased from 17.8 to 11.4 μg/L (results included in Figure 3). In this case, cyclosporine appeared superior to prednisolone in improving supranormal serum Spec fPL concentrations and appetite and likely pancreatic inflammation.

This study has several limitations, mainly due to its retrospective nature. Clinical records were frequently incomplete or not sufficiently detailed, especially concerning clinical signs and severity. Chronic pancreatitis was not confirmed by histopathology which is the current gold standard to diagnose CP [17]. Furthermore, treatment length until follow-up varied substantially, doses differed and tapering occurred at various time points after follow-up, and details on administration of cyclosporine with or without food were unavailable. The administration of cyclosporine with or without food and the different brands and formulations of cyclosporine may have affected bioavailability.

The central question that remains to be answered is if cyclosporine treatment is associated with a protective effect against deterioration of pancreatitis, and subsequent development of DM and EPI in cats or if it is merely associated with a high level of side effects for the cats and their owners. However, the only cats still alive at the time of writing were cats that had remained on cyclosporine treatment. This may suggest a protective effect of cyclosporine treatment in CP.

## 5. Conclusions

Despite the limitations of the study, our results suggest that cyclosporine treatment seems to reduce supranormal serum Spec fPL concentrations in cats with suspected CP and that dose tapering leads to the recurrence of increased serum Spec fPL concentrations. Prospective randomized studies are warranted to investigate the effect of cyclosporine on chronic pancreatic inflammation and clinical outcome of cats with histologically confirmed CP.

## Figures and Tables

**Table 1:** Baseline data, clinical signs and comorbidities at inclusion and follow-up in 19 cats with presumed chronic pancreatitis treated with cyclosporine.

Parameter		Range (Median) or Number of Cats (%)
Age at inclusion (years)	-	6.9–17.5 (11.6)
BW ^1^ at inclusion (kg)	-	3.0–7.2 (5.7)
BCS ^2^ at inclusion	-	3/9–8/9 (6/9)
BW at follow-up (kg)	-	2.7–7.0 (5.6)
BCS at follow-up	-	3/9–7/9 (5/9)
Breed	Domestic short hair	10 (53)
	Mixed breed	2 (11)
	Birman	2 (11)
	Siamese	2 (11)
	Norwegian Forest Cat	2 (11)
	Ragdoll	1 (5)
Gender	Neutered male	16 (84)
	Neutered female	3 (16)
Major clinical signs at	Weight loss	12 (63)
inclusion	Lethargy	9 (47)
	Hyporexia	8 (42)
	Vomiting	8 (42)
	Abdominal pain	6 (32)
	Diarrhea	5 (26)
	Constipation	3 (16)
Comorbidity at inclusion	Chronic enteropathy ^3^	14 (74)
	Chronic kidney disease ^4^	9 (47)
	Diabetes mellitus	6 (32)
	Hepatobiliary disease ^5^	4 (21)

^1^ Body weight. ^2^ Body condition score. Available in 16 cats. ^3^ Based on histopathology (n = 6), subnormal/low-normal serum cobalamin concentration (n = 8) and ultrasonography (n = 2). ^4^ IRIS stage 1 (n = 4) or 2 (n = 5). IRIS staging possible for 15/19 cats. ^5^ Based on recent ultrasonographic findings indicating disease and recent supranormal alanine aminotransferase values (n = 3) or histopathology confirming chronic hepatitis prior to study inclusion (n = 1).

**Table 2:** Histopathology reports of gastrointestinal biopsies (n = 6) and liver biopsies (n = 1) from cats with presumed chronic pancreatitis treated with cyclosporine. Biopsies were collected before study inclusion.

Localization	Results	Severity	Number of Cats
Stomach ^1^	LP ^2^ inflammation	Mild-moderate	5
	LPN ^3^ inflammation	Mild	1
Duodenum ^1^	LP inflammation	Mild-moderate	2
	Fibrosis only	Not specified	1
	No abnormal findings		3
Colon ^1^	LP inflammation	Mild-moderate	6
Liver ^4^	Chronic interface hepatitis	Severe	1

^1^ Sampled endoscopically except one biopsy of the duodenum sampled surgically by laparotomy. The surgical biopsy showed no abnormal findings. ^2^ LP: lymphocytic plasmacytic. ^3^ LPN: lymphocytic plasmacytic and neutrophilic. ^4^ Sampled surgically by laparotomy.

**Table 3:** Diet and concurrent medical treatments at study inclusion and between inclusion and follow-up in 19 cats with suspected chronic pancreatitis treated with cyclosporine. Most of the cats received more than one diet.

Parameter		Number of Cats (%)
Diet at inclusion	CD ^1^: Hydrolyzed	6 (32)
	CD: Single protein	6 (32)
	CD: Highly digestible	4 (21)
	Other CD ^2^	9 (47)
	Commercial raw food	1 (5)
Diet change at follow-up	Other CD ^3^ added	4 (21)
Treatment at inclusion	Corticosteroids	13 (68)
	Cobalamin supplement	2 (11)
	Multistrain probiotic ^4^	2 (11)
	Miscellaneous ^5^	8 (42)
Treatment at follow-up	Corticosteroids ^6^	12 (63)
	Cobalamin supplement	3 (16)
	Multistrain probiotic ^4^	2 (11)
	Psyllium prebiotic	1 (5)
	Miscellaneous ^7^	6 (32)

^1^ CD: Commercial diet. ^2^ Diabetic diet (n = 3), liver diet (n = 1), renal diet (n = 1), other non-veterinary diet (n = 5). ^3^ Non-veterinary diet (n = 3), diabetic diet (n = 1). ^4^ SivoMixx, Ormendes. ^5^ Ondansetron (n = 2), maropitant (n = 2), lactulose (n = 2), metoclopramide (n = 1), prucalopride (n = 1), olsalazine (n = 1). ^6^ Dose unchanged (n = 9) or tapered (n = 3) after inclusion. ^7^ Ondansetron (n = 2), lactulose (n = 2), maropitant (n = 1), mirtazapine (n = 1), olsalazine (n = 1).

**Table 4:** Selected serum chemistry and hematology results at inclusion and from inclusion to follow-up in 19 cats with presumed chronic pancreatitis treated with cyclosporine.

Parameter	Deviation/Time Point	Reference Interval	Range (Median) and Number of Cats
Serum cobalamin			
	Subnormal/low-normal		
	At inclusion	>180 pmol/L	230–263 pmol/L (232) ^1^
			3
	At follow-up	>180 pmol/L	<111 pmol/L
			1
Serum creatinine			
	Increased		
	At inclusion	60–170 µmol/L ^2^	178 µmol/L
			1
		71–212 µmol/L ^3^	221 µmol/L
			1
	At follow-up	60–170 µmol/L ^2^	180–200 µmol/L (195)
			3
Alanine			
aminotransferase			
	Increased		
	At inclusion	0–1.2 µkat/L ^2^	1.5–2.92 µkat/L (1.6)
			3
		<1.4 µkat/L ^4^	1.8 µkat/L
			1
		0.2–2.2 µkat/L ^3^	3.0 µkat/L
			1
	At follow-up	0–1.2 µkat/L ^2^	2.1 µkat/L
			1
Hematocrit			
	Decreased		
	At inclusion	30.3–52.3%	21.4%
			1

^1^ Low-normal values: Within 100 units of the lower reference limit of 180 pmol/L. ^2^ Analyzed at the Evidensia Specialist Animal Hospital, Helsingborg, Sweden. ^3^ Analyzed at Idexx Laboratories, Kornwestheim, Germany. ^4^ Analyzed in-house at a referring clinic.

**Table 5:** Ultrasonographic reports of the pancreas and gastrointestinal tract (GI tract) in cats with presumed chronic pancreatitis treated with cyclosporine.

Localization	Time Point	Findings	Number of Cats
Pancreas			
	At inclusion ^1^	Hypoechoic, broadened, prominent	1
		Hypoechoic, hyperechoic peripancreatic tissue	1
		Hyperechoic areas	1
		No abnormal findings	6
		In total	9
	At follow-up ^1^	Heterogenic	2
		In total	2
GI tract			
	At inclusion ^2^	Reactive lymph nodes ^3^	6
		Prominent/hypertrophic gastric muscularis	1
		Prominent/hypertrophic SI ^4^ muscularis	4
		Prominent/hypertrophic LI ^4^ muscularis	1
		Other ^5^	1
		No abnormal findings	1
		In total	10
	At follow-up ^2^	Reactive gastric lymph nodes	1
		Prominent/hypertrophic SI muscularis	1
		No abnormal findings	1
		In total	2
Hepatobiliary system			
	At inclusion ^6^	Hyperechoic/dense liver parenchyma	3
		Intrahepatic bile duct mineralization	2
		Gallbladder sludge	2
		No abnormal findings	3
		In total	10
	At follow-up ^6^	Hyperechoic liver parenchyma	1
		Gallbladder sludge	1
		In total	2

^1^ In one cat, ultrasonography of the pancreas was performed at both inclusion and follow-up and was abnormal on both occasions. ^2^ In one cat, ultrasonography of the GI tract was performed both at inclusion (abnormal) and at follow-up (no abnormal findings). ^3^ Mesenteric (n = 4) and/or colic (n = 3) lymph nodes. ^4^ SI: small intestine, LI: large intestine. ^5^ Abnormal, elongated and non-peristaltic structure related to the stomach. ^6^ In one cat, ultrasonography of the hepatobiliary system was performed both at inclusion (no abnormal findings) and at follow-up (abnormal).

## Data Availability

Data available on request due to restrictions, e.g., privacy or ethical reasons. As the study is retrospective, no owner consent was signed and the data is therefore not publicly available.
